# Application of Place-Based Methods to Lung Transplant Medicine

**DOI:** 10.3390/ijerph19127355

**Published:** 2022-06-15

**Authors:** Wayne M. Tsuang, Maeve MacMurdo, Jacqueline Curtis

**Affiliations:** 1 Respiratory Institute, Cleveland Clinic, 9500 Euclid Avenue, Cleveland, OH 44195, USA; macmurm@ccf.org; 2 GIS Health & Hazards Lab, Department of Population and Quantitative Health Sciences, School of Medicine, Case Western Reserve University, Cleveland, OH 44106, USA; jxc1546@case.edu

**Keywords:** respiratory, medicine, neighborhood, census tract, organ transplant

## Abstract

Lung transplantation is an increasingly common lifesaving therapy for patients with fatal lung diseases, but this intervention has a critical limitation as median survival after LT is merely 5.5 years. Despite the profound impact of place-based factors on lung health, this has not been rigorously investigated in LT recipients—a vulnerable population due to the lifelong need for daily life-sustaining immunosuppression medications. There have also been longstanding methodological gaps in transplant medicine where both time and place have not been measured; gaps which could be filled by the geospatial sciences. As part of an exploratory analysis, we studied recipients transplanted at our center over a two-year period. The main outcome was at least one episode of rejection within the first year after transplant. We found recipients averaged 1.7 unique residential addresses, a modest relocation rate. Lung rejection was associated with census tracts of predominantly underrepresented minorities or where English was not the primary language as measured by the social vulnerability index. Census tracts likely play an important role in measuring and addressing geographic disparities in transplantation. In a future paradigm, patient spatial data could become an integrated part of real time clinical care to aid in personalized risk stratification and personalized delivery of healthcare.

## 1. Introduction

There has been growing interest in looking beyond the walls of hospitals and clinics to leverage non-clinical health determinants to improve our understanding of individual patient outcomes. To do so, however, requires translational collaboration across multiple disciplines, including between the spatial sciences and clinical medicine. In the case of lung transplantation [LT], given this patient population’s unique risk factors, investigating the link between health and place at a granular level could lead to interventions to help our most vulnerable patients.

Lung diseases are now among the leading causes of death in the United States due to a 30% rise in respiratory morbidity and mortality over the past two decades [1,2]. In response, LT has become the fastest growing segment of organ transplant with an annual volume in the U.S. more than doubling between 2004 and 2017 [3]. LT serves as the definitive intervention for chronic diseases such as COPD, cystic fibrosis, pulmonary fibrosis, pulmonary hypertension [4,5,6], and for end stage lung disease caused by COVID-19 [7,8,9,10]. However, there remains a key limitation: worldwide median patient survival after LT remains only 5.5 years, which has remained stagnant since the beginnings of modern transplantation in the 1980s and is merely half the survival seen in heart, kidney, or liver transplantation [11].

While many factors play a role in survival after LT, we know our lungs are especially vulnerable organs due to being directly and continuously exposed to our surroundings through inhalation of air particulate matter, dust, mold, and susceptible to infection. In addition, this vulnerability is exacerbated by immunosuppression medications transplant recipients must take daily for life to prevent the immune system’s rejection of the donor lungs. Lung function after LT can be impacted by social vulnerabilities as well such as through low adherence to transplant medications, missed healthcare visits, or loss of health insurance [12,13,14]. 

A gap in knowledge exists on the role of place—namely on the role of environmental and social vulnerabilities. This gap is due to a missing framework within transplant medicine from which to rigorously study place. The Geographic Information Sciences (GISc) provides one approach to study place—which we define broadly as an area, neighborhood, or community with meaning to a person. Place can be quantified at multiple levels —ranging from zip-codes to smaller units such as census tracts. While familiar to the spatial sciences and public health, census tracts remain comparatively unfamiliar and unutilized in clinical medicine.

### 1.1. Lung Transplantation and Geography

In lung transplant, national data have shown there are wide geographic differences in outcomes. The median LT survival in the U.S. can range from a low of 4.6 years to a high of 6.4 years depending on which one of 11 geographic regions in the U.S. a patient was from; resulting in a geography dependent relative risk of survival of 39% (*p* < 0.001) [15]. There is a need to understand place-based contextual factors exacerbating the geographically disparate and short survival of LT recipients in order to more precisely risk stratify patients and identify opportunities for intervention.

Traditionally, clinical medicine has relied on zip codes as a longstanding form of geolocation as they are easily drawn from the patient records of a health system or collected for large clinical disease registries. Examples include the registries of the American Heart Association, Cystic Fibrosis Foundation, or the United Network for Organ Sharing [16,17,18]. As a result, zip code has been frequently used to assess the socioeconomic position of an area.

**Figure 1:**
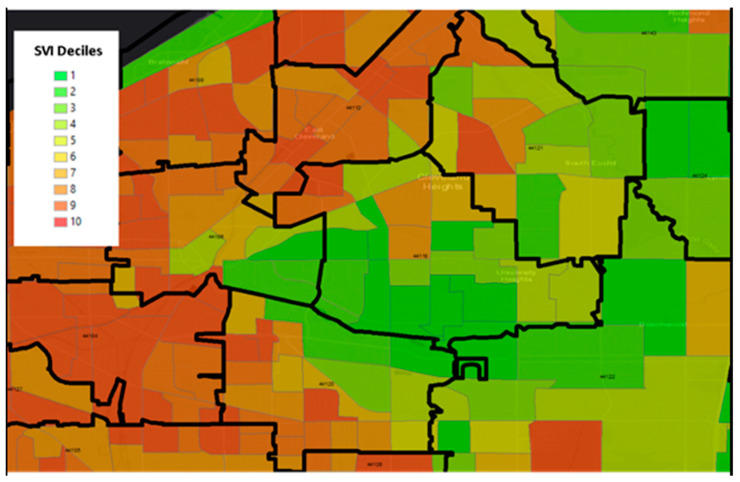
Example of zip code (black outline) vs. census tract (green = low social vulnerability to red = high social vulnerability). CDC Social Vulnerability Index (SVI) is represented as census tracts classified by deciles (green = low social vulnerability to red = high social vulnerability) and zip codes are represented with black outlines.

Figure 1 is an example that demonstrates the differences in size between Zip Code Tabulation Areas (ZCTAs), which are a U.S. Census geographic unit designed to approximate a zip code, and census tracts in the east region of Cleveland, Ohio. The census tracts display the Center for Disease Control and Prevention’s publicly available Social Vulnerability Index (SVI). The map is centered on zip code 44106, the location of Case Western Reserve University. There are no zip codes with homogeneous social vulnerability within their boundaries, providing visual evidence that the ZCTA unit of aggregation is suboptimal for detecting geographically granular health-place relationships. As a result, reliance on zip-code level socioeconomic status or disparity data may not adequately capture the individual patient environment. Census tracts, on the other hand, provide more rigor and granularity and serve as a link to a wide range of publicly available datasets representing multiple areas such poverty levels or environmental exposures. In this exploratory analysis we leverage the utility of the census tract.

### 1.2. Solving Methodologic Gaps

Addressing the knowledge gap between place and health requires us to solve two troublesome methodologic gaps. First, most existing investigations of social-environment relationships measure exposures based on only one place: the patient’s home address at the time of a certain event (e.g., at diagnosis of a disease or at the time of a procedure). This approach assumes that the home address is stable, and that the most meaningful exposures for health outcomes occur at this location. Second, these investigations also assume neighborhood conditions surrounding this home address are stable and are often measured at only one scale (e.g., a zip code). However, these are both problematic assumptions. People’s mobility is dynamic, spanning timeframes from each day to over the life course and the environment to which people are exposed is also dynamic (e.g., exposure to airborne particulate matter can vary daily, seasonally, etc.,). Despite the serious implications these measurement errors have for scientific knowledge and clinical translation, they have received little attention. Thus, can methods used to measure patient context and mobility be improved and translated to predict clinical outcomes more precisely? Answering this question requires an approach addressing place and time.

Studying place attempts to address the question—where is the patient? Census tracts can be a valuable tool in this process, allowing linkage to important data we refer to as “geomarkers”, as previously described by Beck et al. [19,20,21]. Similar to how biomarkers (e.g., vital signs or blood tests) inform clinical decisions, geomarkers are quantitative data at the census tract level that more precisely stratify risk and exposure within a patient’s community. This information can be utilized to inform changes in clinical care. Similarly, when considering patient place, we also need to consider duration of residence—“how long has the patient lived at a location”. Long time residence in a location may be associated with a higher risk of location-specific exposure—but may also result in a stronger sense of community, and a better understanding of the resources present within that location. Similar to how biomarkers are collected and stored in biobanks, the collection of patient geomarkers over time enables the creation of a “geobank”. We define a geobank as an accounting of individual residential address history with the inclusion of time at each address. This answers a problematic assumption of prior GISc work that patient location is static and allows for more precise discrimination of environmental and community level vulnerabilities.

### 1.3. Electronic Health Records: A Data Source for Spatial Research

Electronic Health Records (EHRs) are ubiquitous in the U.S. healthcare system and have streamlined record keeping and enabled real time measurement of a wide range of metrics. A key application for the spatial sciences is the recording of residential addresses and address history. As part of the regular patient registration process for any outpatient or inpatient visit, basic identifiers are used such as patient name and birthdate, as well as verification of home address. Accurate addresses are important to hospitals so that appointment reminders, test results, or medical bills can be mailed to the correct address. As a result, many EHRs may have a rich database of patient reported residential address history and changes in address can indicate the time lived at each address. The collection of address history has the potential to reduce misclassification bias when assigning an exposure to a location, as the most recent address on record may not be the address where the patient spent the longest amount of time. 

A limitation of the EHR is that address history is only captured when patients interface with the health system. For example, a patient new to a community and new to a health system may not have a longitudinal record of address history. Moreover, EHRs are unique and often customized to a specific hospital system which are protected by cybersecurity firewalls. A patient seen and treated at two different health systems within the same city could have two separate electronic medical charts. Despite these limitations, there are growing EHR linkages among separate health systems to more easily facilitate the secure sharing of patient medical information. We hypothesized that spatial methods used to understand time and place could be applied to EHR data in order to better risk stratify our patient population. 

## 2. Materials and Methods

In this exploratory analysis, we identified recipients of LT at our center between 1 January 2016 and 31 December 2017. Clinical data were extracted from the institutional electronic health record. Native lung disease was defined using definitions from the United Network for Organ Sharing (UNOS)-the national organization charged with organ allocation policy in the United States and categorized as: group A—obstructive disease (primarily emphysema/COPD), group B—vascular disease (primarily pulmonary hypertension), group C—cystic disease (primarily cystic fibrosis), or group D—fibrotic disease (primarily pulmonary fibrosis) [22]. Patients < 18 years at time of transplant, underwent multi-organ transplant, or had invalid address were excluded. We also excluded recipients who moved from outside the U.S. solely for transplant or used P.O. Box addresses. Geobanking: Residence was defined as a self-reported recipient address used to register in the electronic health record which was updated or verified at any clinical encounter. The number of unique addresses per recipient were recorded. Geomarking: We identified the latitude and longitude of the residential address of each patient in order to geocode the census tract. The tract was then linked to the Center for Disease Control and Prevention’s Social Vulnerability Index (SVI), which scores all U.S. census tracts in four domains: Socioeconomic status, Household composition, Race/ethnicity/language, and Housing/transportation. The SVI ranges from 0 to 1, with higher values indicating greater vulnerability [23].

Our primary outcome was whether recipients experienced ≥1 episode of moderate grade biopsy-proven acute lung rejection (grade ≥ A2) [24]. The diagnosis of lung rejection is made by a specialized pathologist reviewing a lung biopsy and applying diagnostic criteria established by the International Society of Heart and Lung Transplantation (ISHLT) (25). Episodes of acute rejection can occur in up to 40% of patients within the first year after transplant, and are a leading risk factor for chronic rejection—often referred to as the Achilles’ heel of LT as it is the leading cause for LT mortality [25]. We focused on episodes > 3 months after transplant where lung rejection was likely from place-based factors and less likely peri-operative related issues. Patients do not necessarily require hospitalization for the treatment of rejection.

An ANOVA was used to test for differences between SVI categories using R (R Core Team, 2020). Geocoding was through ArcGIS 10.7.1 (Redmond, WA, USA). This study was approved by the Cleveland Clinic Institutional Review Board (#20-804). 

## 3. Results

There were 232 LTs who met inclusion criteria. From this cohort 65 (28.0%) had COPD, 5 (2.2%) had pulmonary hypertension, 22 (9.5%) had cystic fibrosis/bronchiectasis, and 140 (60.3%) had pulmonary fibrosis (Table 1). The most common indication for transplant was pulmonary fibrosis (60.3%), and 31.9% were female. We identified 389 unique addresses through the health system electronic health record. Unique addresses per recipient ranged from 1 to 11, with an average 1.7 addresses per recipient and a median of 1. Among recipients with biopsy-proven moderate grade early rejection, there was an average of 1.84 addresses (possibly more mobility), while those without rejection averaged 1.59 addresses (possibly less mobility), though this difference was not significant. A total of 45 patients experienced moderate grade rejection. About 31 were within 3 months of transplant and 14 were >3 months. Among patients with >3 months rejection, a one-way ANOVA revealed that there was a statistically significant difference in mean score between at least two groups (F(3, 48) = [4.5669], *p* = 0.007) where census tracts predominantly of underrepresented minorities or where English is not the predominant language (SVI: 0.64) when compared to socioeconomic status (SVI: 0.31), household composition (SVI: 0.36), or housing/transportation (SVI: 0.37) (*p* = 0.007) (Table 2).

## 4. Discussion

For this exploratory analysis, we successfully merged recipient residential history with clinical data, and found that recipients of LT have a modest relocation rate. The relocation rate of other organ transplant types is unknown. Frequent re-location may signify housing and economic instability, and portend worse health outcomes through the unintended fracturing of continuous healthcare. This information is essential for place-based studies to address geographic disparities and reduce misclassification bias. While our preliminary analysis did not identify a difference by risk for early rejection and may have been limited by sample size, other clinical outcomes warrant investigation. Further studies can also assess the implications of relocation toward a census tract with higher SVI or lower SVI. Further qualitative studies may consider asking patients the reasons for relocation and if they felt relocation impacted their health.

We also successfully leveraged U.S. census tracts to enable linkage to an important non-clinical SVI dataset. We found that a census tract-based geomarker representing underrepresented minorities or where English was not the predominant language was associated with the risk for biopsy-proven acute cellular lung rejection—a serious but mutable risk factor for lung function loss and early mortality. The reasons for this require further investigation but could be related to frequency of healthcare visits, access to transplant and non-transplant healthcare, and in communities where English is not the dominant language fully accessing transplant-related education material which guides patients through lifestyles recommendations. Because acute lung rejection is a mutable risk factor, and a patient’s SVI is readily identifiable, this approach could be quickly translated to individualized clinical care through shortened outpatient follow-up intervals or augmented immunosuppression to improve outcomes for at-risk patients. Census tracts likely play a promising role in identifying and addressing geographic disparities in clinical transplantation. 

### 4.1. Limitations and Considerations

**Figure 2:**
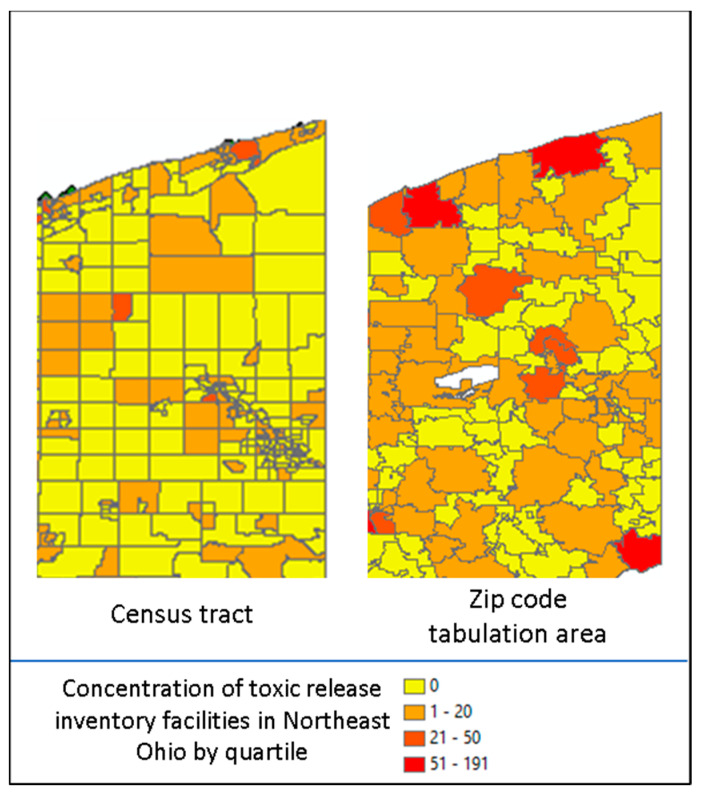
Example of Modifiable Area Unit Problem in Northeast Ohio.

There were limitations to our approach. In creating a geobank of address history, the reasons for relocation were unknown, patient home addresses were self-reported, and residential history prior to becoming a patient at our health system was unknown. Additionally, two key conceptual issues must be considered with geocoded data. The first is the Modifiable Area Unit Problem (MAUP) which refers to when point-based measures of spatial phenomena are aggregated into districts. The resulting summary statistics (e.g., rates, densities) are influenced by both the shape and scale of the spatial unit and could be a source of statistical bias [26]. As an example, in Figure 2, displayed are the concentration of toxic release inventory facilities by Ohio census tract (left) as compared to Ohio zip code tabulation area (right) by quartile (red = high concentration and yellow = low concentration). The spatial unit used suggests differing distributions [27]. Alternative approaches are to use spatial sensitivity analysis with a variety of areal units to estimate the uncertainty of correlation, or to use the most granular spatial unit available as we did by leveraging the census tract. The second is the ecological fallacy, which is the inference about the nature of individuals deduced from groups or surroundings [28]. An example is attempting to deduce an individual patient’s blood pressure from the average blood pressure of a county population, where group level findings may not be present at the individual level. Acknowledging and applying these limitations to the interpretation of geocoded data adds to the rigor of the research.

### 4.2. Privacy

The analysis of geocoded patient data should be accompanied by strict data safeguards. Though the geographic mapping of clinical patient data is powerful, it should be done in a way to prevent the unintentional loss of patient anonymity. With the electronic media and the Internet providing instant access to personal information, the risk for reverse engineering a map to identify an individual patient is very real [29]. There are approaches to geo-masking patient data while still producing an impactful map, if one is needed at all.

### 4.3. Clinical Applications

Address history and census tract level vulnerability raise several potential translational applications through risk stratification and cluster identification. For example: (a) At risk patients could be followed up in outpatient clinic at shorter time intervals to ensure closer attention by a clinical team; (b) clinicians could personalize medication use through more proactive use of antibiotics or antivirals to prevent pneumonias; (c) if a clinician knew a patient resided next to a major highway or large factory where air pollution levels were elevated, this could lead to earlier interventions such as screening for lung diseases, counseling the patient on how to reduce exposure to air pollution, or helping the patient obtain a home air filtration system; (d) cluster identification: there is an additional need for real time chronic disease monitoring in the community which could be met through GISc [30,31]. Chronic disease detection in a community may take months or years to observe, which pales in comparison to the pace of detection in more acute settings. For example, hospitals frequently track the number of nosocomial infections within a hospital on a week to week basis. An uptick in cases in a specific ward would lead to rapid responses and interventions to reduce the infection rate. Similarly, serious epidemic infections in the community, such as COVID-19, have been closely tracked, mapped, and reported at a near daily pace to inform communities on risk levels and hospital preparedness. However, less acute diseases such as diabetes, hypertension, pneumonias, or lung diseases have not been surveilled as closely. Imagine if an EHR could track the number of cases of COPD within a census tract that an individual patient is from and display this in real time on a computer screen during the outpatient visit. Then, when reviewing clinical data, the physician also sees the spatial information which immediately informs her pre-test probability and decision-making on the screening, testing, and counseling for potential COPD in this individual patient. This workflow still requires a framework for implementation and use, but would be a major advancement for both the clinical and spatial sciences. Perhaps in the future we may have a whole new field unto itself, which transforms the Geographic Information Sciences into the Geographic Translational Sciences. In this new paradigm, geomarkers and geobanks would become an integrated part of real time clinical care and an essential vital sign to aid in the personalization of lung transplant healthcare.

## 5. Conclusions

Our long-term goal is to improve the suboptimal survival of LT recipients, and to ensure this improvement occurs regardless of place. We explored the possibility of creating a geobank where residential history was codified as both current and previous home addresses and employing geomarkers to understand clinical outcomes. Understanding both the location and duration of residence in a place is a necessary link to understanding socioeconomic, geographic, and environmental disparities which can influence health outcomes.

## Figures and Tables

**Table 1:** Demographics.

Category	n (%) or Average
	n = 232
Female	74 (31.9)
Average age in years at transplant	59
Race	
White	202 (87)
Black	13 (6.9)
Other	17 (7.3)
Reason for transplant	
COPD	65 (28)
Cystic fibrosis	22 (9.5)
Pulmonary hypertension	5 (2.2)
Pulmonary fibrosis	140 (60.3)
Average Body Mass Index (kg/m^2^)	25.8
Average height (centimeters)	170.3

**Table 2:** Centers for Disease Control Social Vulnerability Index.

	Socioeconomic Status	Household Composition	Underrepresented Minorities	Housing/Transportation	*p*	F-Stat
**Early lung rejection**	0.36	0.38	0.47	0.42	0.48	0.83
**Late lung rejection**	0.31	0.36	0.64	0.37	0.007	4.57

## Data Availability

The data presented in this study are available on request from the corresponding author.
